# B and T Cells Driving Multiple Sclerosis: Identity, Mechanisms and Potential Triggers

**DOI:** 10.3389/fimmu.2020.00760

**Published:** 2020-05-08

**Authors:** Jamie van Langelaar, Liza Rijvers, Joost Smolders, Marvin M. van Luijn

**Affiliations:** ^1^Department of Immunology, MS Center ErasMS, Erasmus MC, University Medical Center, Rotterdam, Netherlands; ^2^Department of Neurology, MS Center ErasMS, Erasmus MC, University Medical Center, Rotterdam, Netherlands; ^3^Neuroimmunology Research Group, Netherlands Institute for Neuroscience, Amsterdam, Netherlands

**Keywords:** Th1/Th17, T-bet^+^ B cells, CD8^+^ T cells, Epstein-Barr virus, genetic risk, transmigration, germinal center, IFN-γ

## Abstract

Historically, multiple sclerosis (MS) has been viewed as being primarily driven by T cells. However, the effective use of anti-CD20 treatment now also reveals an important role for B cells in MS patients. The results from this treatment put forward T-cell activation rather than antibody production by B cells as a driving force behind MS. The main question of how their interaction provokes both B and T cells to infiltrate the CNS and cause local pathology remains to be answered. In this review, we highlight key pathogenic events involving B and T cells that most likely contribute to the pathogenesis of MS. These include (1) peripheral escape of B cells from T cell-mediated control, (2) interaction of pathogenic B and T cells in secondary lymph nodes, and (3) reactivation of B and T cells accumulating in the CNS. We will focus on the functional programs of CNS-infiltrating lymphocyte subsets in MS patients and discuss how these are defined by mechanisms such as antigen presentation, co-stimulation and cytokine production in the periphery. Furthermore, the potential impact of genetic variants and viral triggers on candidate subsets will be debated in the context of MS.

## Introduction

In multiple sclerosis (MS) patients, pathogenic lymphocytes are triggered in the periphery to infiltrate the central nervous system (CNS) and cause local inflammation and demyelination. Anti-CD20 therapy has recently been approved as a novel treatment modality for MS (1–3). Although this underscores the fact that B cells play a key role in MS, the exact triggers, subsets and effector mechanisms contributing to the disease course are incompletely understood. The impact of this therapy on the antigen-presenting rather than the antibody-producing function of B cells in MS indicates that their interaction with T cells is an important driver of the pathogenesis (1, 4). Alterations in cytokine production, co-stimulation and antigen presentation most likely contribute to the development of pathogenic B and T cells that are prone to enter the CNS (4, 5). Such mechanisms might be influenced by the interplay between genetic and environmental risk factors (6). The major *HLA-DRB1^∗^1501* locus accounts for 30% of the overall risk (6) and has been shown to promote B cell-mediated induction of brain-infiltrating T helper (Th) cells in MS patients (4). Besides for *HLA-DRB1^∗^1501*, other genetic risk variants that have been identified in the past decades also appear to potentiate B and Th cell activation, a feature that is shared amongst several autoimmune disorders (7). Furthermore, infectious triggers such as the Epstein-Barr virus (EBV) alter their function and reactivity in MS (5, 6, 8, 9). The current view is that transmigration of lymphocyte subsets into the CNS signifies relapsing disease, while compartmentalized CNS inflammation, as seen during disease progression, seems to be driven by tissue-resident populations (10, 11). Since there is a clear association of relapse occurrence and radiological disease activity early in MS with the severity of disability progression later in MS (12), it is crucial to understand what motivates these cells to invade the CNS and why these cells instigate local pathology in MS patients.

In this review, we will discuss which and how brain-infiltrating lymphocyte subsets can contribute to MS pathogenesis. These pathogenic events are characterized by: (1) peripheral escape of pathogenic B cells from T cell-mediated control, (2) mutual activation of pathogenic B and T cells within peripheral germinal centers, and (3) re-activation of infiltrating B and T cells within the CNS. We will use current knowledge to consider the extent to which genetic and viral triggers may drive these pathogenic events in MS.

## Impaired T Cell-Mediated Control of Pathogenic B Cells in MS

B and T cells closely interact in secondary lymphoid organs to generate an optimal immune response against invading pathogens. Within follicles, B cells recognize antigens via the highly specific B-cell receptor (BCR), resulting in internalization, processing and presentation to T cells. This mechanism is unique and tightly coordinated involving five consecutive and interdependent steps: (1) B-cell receptor signaling, (2) actin remodeling, (3) endosomal formation and transport, (4) HLA class II synthesis and trafficking to specialized late endosomes (i.e., MIICs), and (5) antigen processing and loading onto HLA class II molecules for presentation to CD4^+^ Th cells (13, 14). Through their interaction with Th cells, germinal center (GC)-dependent and -independent memory B cells are formed, a process that is governed by the strength of the HLA/peptide signal (15). GC B cells respond to interleukin (IL)-21-producing follicular Th (Tfh) cells to develop into class-switched (IgG^+^) subsets or antibody-producing plasmablasts/plasma cells (15, 16). Memory B cells, in return, specifically trigger Th effector subsets that help CD8^+^ cytotoxic T cells (CTLs) to kill the infected cell (17). In MS, this crosstalk between B and T cells is likely disturbed, eventually causing pathogenic instead of protective immunity. This may already start during selection of naive autoreactive B cells in the periphery.

**FIGURE 1:**
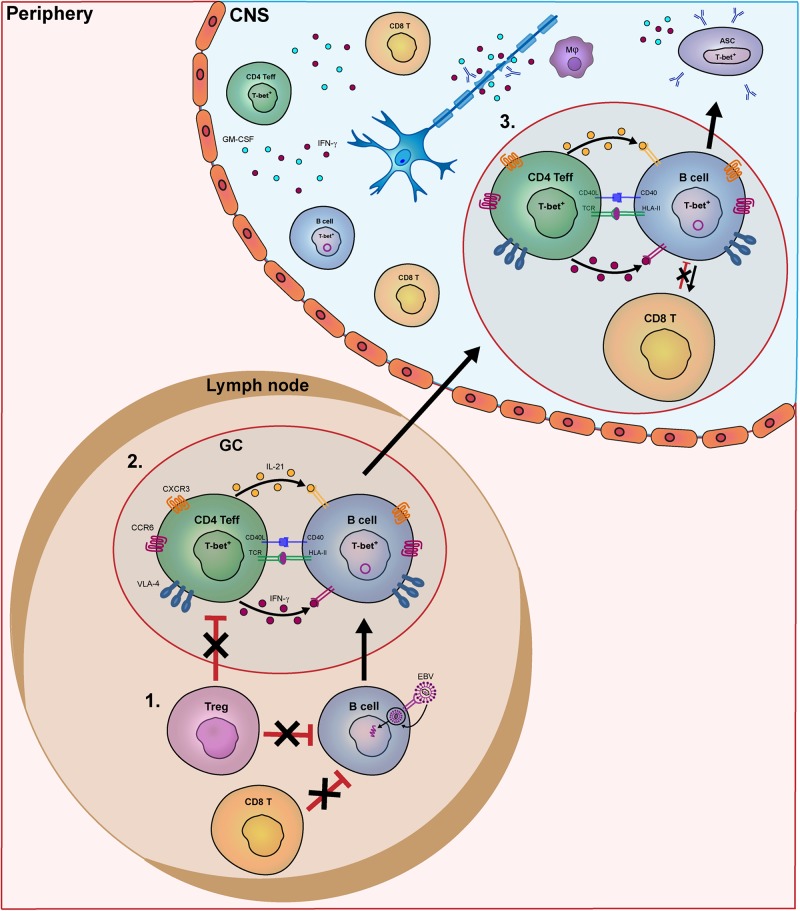
Model of the key pathogenic events involving human B- and T-cell subsets driving MS disease activity. In MS patients, B- and T-cells interact in the periphery and central nervous system (CNS) to contribute to disease pathogenesis. In this model, we put forward three important meeting points of pathogenic B and T cells that drive the disease course of MS. In secondary lymphoid organs, B-cell tolerance defects in MS patients allow EBV-infected B cells to escape from suppression by CD8^+^ and T regulatory (Treg) cells **(1)**. Subsequently, these activated B cells enter germinal centers (GCs) and interact with follicular Th cells to further differentiate into pathogenic memory B cells. Under the influence of IFN-γ and IL-21, B cells develop into T-bet-expressing memory cells, which in turn activate Th effector cells such as Th17.1 **(2)**. These subsets are prone for infiltrating the CNS of MS patients by distinct expression of chemokine receptors (CXCR3, CCR6), adhesion molecules (VLA-4) as well as pro-inflammatory cytokines. **(3)** Within the CNS, IFN-γ-, and GM-CSF-producing T cells and T-bet^+^ memory B cells probably come into contact in follicle-like structures, resulting in clonal expansion inflammation and demyelination. T-bet^+^ memory B cells further differentiate into plasmablasts/plasma cells to secrete high numbers of potentially harmful antibodies (oligoclonal bands).

Normally, after removal of the majority of B-cell clones expressing polyreactive antibodies in the bone marrow (central tolerance), surviving autoreactive B cells are kept in check by peripheral tolerance checkpoints (18). In contrast to most other autoimmune diseases, only peripheral and not central B-cell tolerance checkpoints are defective in MS, which coincides with increased frequencies of naive polyreactive populations in the blood (18–21). Although the exact cause is currently unknown, the escape of pathogenic B cells from peripheral control may be related to (1) chronic T-cell stimulation and (2) T cell-intrinsic defects (see Figure 1).

Epstein-Barr virus is one of the most thoroughly investigated pathogens regarding T-cell responses in MS. Many theories have been proposed how EBV can influence MS pathogenesis (9). One hypothesis is that, due to the chronic nature of this infection, continuous antigen presentation by B cells leads to functionally impaired, so-called “exhausted” T cells (8, 22). This, together with the impact of HLA and other risk alleles (23), may result in inappropriate T cell-mediated control of EBV-infected (pathogenic) B cells. Consistent with this, peripheral CD8^+^ CTLs show decreased responses to EBV and not to cytomegalovirus antigens during the MS course (8). EBV antigens can also induce IL-10-producing CD4^+^ T regulatory cells (Tregs) capable of suppressing effector T-cell responses to recall antigens (24), as seen for other persistent viral infections such as lymphocytic choriomeningitis virus (25, 26). However, forkhead box P3 (FOXP3^+^) Tregs have also been described to control infections (27), suggesting that additional T cell-intrinsic defects are involved. For example, Treg populations that are enriched in MS patients produce increased levels of interferon gamma (IFN-γ), express reduced levels of FOXP3 and have defective suppressive activity *in vitro* (28). This is not only accompanied with less suppression of effector T cells (29, 30), but possibly also with impaired removal of pathogenic B cells, as described for other autoimmune diseases (18, 31, 32). The direct impact of Tregs on B cells in MS patients is still unknown. Treg function may be altered by variation in *IL2RA* and *IL7RA*, two known MS risk loci (33, 34). FOXP3 correlates with IL-2 receptor (IL-2R) as well as IL-7 receptor (IL-7R) expression in Tregs (35). It can thus be expected that *IL2RA* and *IL7RA* (33, 34), but also *BACH2* (36) variants impair Treg development in MS. This may even influence FOXP3- and IL-2R-expressing CD8^+^ T cells, which can suppress pro-inflammatory CD4^+^ Th cells (37) and are reduced in the blood during MS relapses (38–40).

## The Germinal Center as a Powerhouse of Pathogenic B- and TH-Cell Interaction in MS

### Th Cells as Inducers of Pathogenic Memory B Cells

**FIGURE 2:**
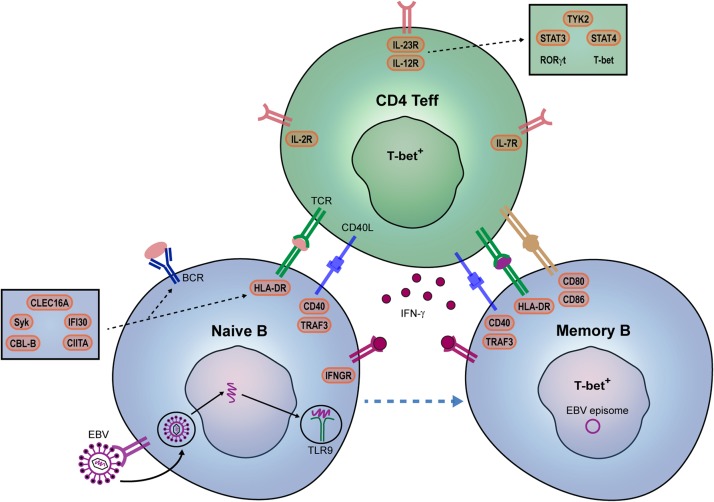
Potential contribution of EBV and genetic risk factors to pathogenic B- and Th-cell development in MS patients. IFN-γ is a key player in autoreactive B- and Th-cell interaction and autoimmune germinal center (GC) formation in mice. In MS, we propose that EBV infection together with specific genetic risk variants promote the IFN-γ-mediated interplay between B and T cells within GCs. EBV directly infects naive B cells and mimic GC responses. EBV DNA can also bind to TLR7/9, and together with IFN-γ, induces T-bet^+^ memory B cells. Their interplay may be additionally stimulated by both B cell-intrinsic (IFN-γ sensitivity: *IFNGR2*; B cell receptor-antigen uptake: *CBLB*, *SYK*, *CLEC16A*; HLA class II pathway: *CLEC16A*, *CIITA*, *IFI30*; co-stimulation: *CD80, CD86*) and Th cell-intrinsic (surface receptors: *IL2RA*, *IL7RA*, *IL12RB1*; downstream molecules: *TYK2*, *STAT3*, *STAT4*) genetic risk variants. IL12R/IL-23R complexes trigger JAK2/STAT3-dependent RORγt and TYK2/STAT4-dependent T-bet expression in Th effector cells.

After their escape from peripheral tolerance checkpoints, naive B cells likely interact with Th cells in GCs to eventually develop into memory populations potentially capable of infiltrating the MS brain (Figure 1). Little is known about how peripheral effector Th cells mediate the development of such pathogenic B cells in MS patients. In GCs of autoimmune mice, autoreactive B cells are triggered by Tfh cells producing high levels of IFN-γ (16). IFN-γ induces the expression of the T-box transcription factor T-bet, which upregulates CXC chemokine receptor 3 (CXCR3), elicits IgG class switching and enhanced antiviral responsiveness of murine B cells (41–43). Recently, we found that B cells from MS patients preferentially develop into CXCR3^+^ populations that transmigrate into the CNS (44). The IFN-γ receptor (IFNGR) and downstream molecule signal transducer and activator of transcription (STAT)1 in B cells are major determinants of autoimmune GC formation in mice (45, 46). After ligation of the IFNGR, STAT1 is phosphorylated, dimerizes and translocates into the nucleus to induce genes involved in GC responses, such as T-bet and B-cell lymphoma 6 (BCL-6) (16, 47). Although IFN-γ-stimulated B cells of MS patients show enhanced pro-inflammatory capacity (44, 48), it is unclear whether alterations in the IFN-γ signaling pathway contribute to the development of T-bet^+^ B cells infiltrating the CNS. Interestingly, a missense SNP in *IFNGR2* has been found in MS, which may alter their development (49, 50). Another target gene of the IFN-γ pathway is *IFI30*, which encodes for the IFN-γ-inducible lysosomal thiol reductase (GILT) and is considered one of the causal risk variants in MS (7). GILT is a critical regulator of antigen processing for presentation by HLA class II molecules (51–53). Together, these findings point to T-bet-expressing B cells as potent antigen-presenting cells that are highly susceptible to triggering by IFN-γ-producing Th effector subsets in MS (44, 54) (Figure 2).

Epstein-Barr virus may be an additional player in the formation of T-bet-expressing B cells. In mice, persistent viral infections sustain the development of these types of B cells, in which T-bet enhances their ability to recognize viral and self-antigens (41, 55). EBV is hypothesized to persist latently in pathogenic B cells and mimic T-cell help for further differentiation in GCs (5, 22, 56, 57). During acute infection, EBV uses a series of latency programs that drive B cells toward a GC response in an antigen-independent manner. Latent membrane protein (LMP)2A and LMP1 resemble signals coming from the BCR and CD40 receptor (56, 57). In addition to their regulation of GC responses independently of T-cell help (58), recent evidence implicates that LMP2A and LMP1 can synergize with BCR and CD40 signaling as well (59). Interestingly, downstream molecules of the BCR (e.g., Syk, CBL-B) and CD40 receptor (e.g., TRAF3) are genetic risk factors for MS (23, 60), therefore potentially cooperating with these latent proteins to enhance pathogenic B-cell development (Figure 2). This is supported by the binding of LMP2A to Syk in B cells and their escape from deletion in GCs of transgenic mice (61). Alternatively, pathogenic B cells can be induced via pathogen-associated TLR9, which binds to unmethylated CpG DNA and further integrate with BCR, CD40, and cytokine signals (62–65). Moreover, pathogenic B-cell responses in systemic autoimmune diseases such as systemic lupus erythematosus are enhanced after IFN-γ and virus-mediated induction of the T-bet (45, 55, 64, 65). In MS patients, TLR9 ligation is also a major trigger of pro-inflammatory B cells (48) and crucial for the differentiation of T-bet-expressing IgG1^+^ B cells during IFN-γ- and CD40-dependent GC-like cultures *in vitro*. Thus, under influence of specific genetic factors, EBV might join forces with IFN-γ-producing Th cells to stimulate pathogenic (T-bet^+^) GC B cells both in a direct (via infection and persistence in pathogenic subsets) and indirect (via TLR7/9) fashion in MS (Figure 2).

### B Cells as Inducers of Pathogenic Memory TH Cells

Synchronously, within peripheral GCs, T-bet-expressing memory B cells are ideal candidates to trigger IFN-γ-producing, CNS-infiltrating Th cells in MS (Figure 1). In both mice and humans, T-bet promotes the antigen-presenting cell function of B cells. This may be related to the impact of EBV infection on B cell-intrinsic processing and presentation of antigens such as myelin oligodendrocyte glycoprotein (MOG) (5). The potent antigen-presenting cell function of B cells in MS patients is further reflected by the effective use of anti-CD20 therapy. This therapy does not affect antibody serum levels, but significantly reduces pro-inflammatory Th-cell responses in MS, both *ex vivo* and *in vivo* (1). CD20 was found to be enriched on IFN-γ-inducible T-bet-expressing IgG^+^ B cells in MS blood (44), pointing to this pathogenic subset as an important therapeutic target. Furthermore, genetic changes in HLA class II molecules, as well as costimulatory molecules [e.g., CD80 (66, 67) and CD86 (68)], may additionally enhance Th cell activation by such memory B cells (Figure 2). HLA class II expression on murine B cells was reported to be indispensable for EAE disease onset (69, 70). The *in silico* evidence that autoimmunity-associated HLA class II molecules have an altered peptide-binding groove (71, 72), together with the potential role of several minor risk variants in the HLA class II pathway [e.g., *CIITA, CLEC16A, IFI30* (Figure 2)], insinuates that antigens are differently processed and presented by B cells (4, 5). This is supported by the increased ability of memory B cells to trigger CNS-infiltrating Th cells in MS patients carrying *HLA-DRB1^∗^1501* (4). These CNS-infiltrating T cells induced by B cells showed features of both Th1 and Th17, therefore representing highly pathogenic subsets. Such subsets are characterized by master transcription factors T-bet and RORγt (73, 74), of which the latter is involved in the co-expression of IL-17 and GM-CSF in mice but not in humans (75, 76). GM-CSF is an emerging pro-inflammatory cytokine produced by Th cells in MS (33, 75, 77). Our group recently revealed that a Th subset producing high levels of IFN-γ and GM-CSF, but low levels of IL-17, termed Th17.1, plays a key role in driving early disease activity in MS patients (78). Proportions of Th17.1 cells were reduced in the blood and highly enriched in the CSF of rapid-onset MS patients. In addition, Th17.1 cells and not classical Th1 and Th17 cells accumulated in the blood of MS patients who clinically responded to natalizumab (anti-VLA-4 mAb). The increased pathogenicity of Th17.1 is further exemplified by their high levels of multidrug resistance, anti-apoptotic and cytotoxicity-associated genes *ABCB1* (MDR1), *FCMR* (TOSO) and *GZMB* (granzyme B), respectively (78–81). Th17.1 cells also show pronounced expression of the IL-23 receptor (IL-23R) (78), which is essential for maintaining the pathogenicity of Th17 cells during CNS autoimmunity (82). IL-23 signals through the IL-23R and IL-12 receptor beta chain (IL-12Rβ1), resulting in JAK2-mediated STAT3 and TYK2-mediated STAT4 phosphorylation, and thereby inducing RORγt and T-bet, respectively (83). *IL-12RB1*, *TYK2*, *STAT3*, and *STAT4* are known genetic risk variants and thus may directly induce Th effector cells in MS (Figure 2). In addition to its potential effect on Tregs (see above), MS-associated risk variant *IL-2RA* enhances GM-CSF production by human Th effector cells (33). To confirm the influence of these and other risk loci (84) on the induction of pathogenic Th cells such as Th17.1 in MS, functional studies need to be performed in the near future.

The increased pathogenicity of Th effector cells may additionally be skewed by IL-6-producing B cells (85, 86), which have been shown to trigger autoimmune GC formation and EAE in mice (87, 88). Blocking of IL-6 prevents the development of myelin-specific Th1 and Th17 cells in EAE (89). The IL-6-mediated resistance of pathogenic Th cells to Treg mediated suppression in MS (90, 91) further links to the abundant expression of anti-apoptotic gene *FCMR* in Th17.1 (78, 92). Intriguingly, B cell-derived GM-CSF can be an additional cytokine driving pathogenic Th cells in MS patients by inducing pro-inflammatory myeloid cells (93). Although the causal MS autoantigen is still unknown, previous work implies that B cell-mediated presentation of EBV antigens at least contributes to pathogenic Th-cell induction (5, 94). As mentioned above, antiviral CD8^+^ CTLs can become exhausted during persistent viral infections. Normally, this mechanism is compensated by the presence of cytotoxic CD4^+^ Th cells, which keep these types of infections under control (95). Such Th populations have been associated with MS progression (96) and are also formed after EBV infection, producing high levels of IFN-γ, IL-2, granzyme B, and perforin (97, 98). Similarly, EBV- and myelin-reactive Th cells from MS patients produce high levels of IFN-γ and IL-2 (6) and strongly respond to memory B cells presenting myelin peptides (99). These studies indicate that the involvement of EBV-infected B cells, especially those expressing T-bet (see section “Th Cells as Inducers of Pathogenic Memory B Cells”), in activating Th effector cells with cytotoxic potential (78, 100, 101) deserves further attention in MS.

## Reactivation of CNS-Infiltrating B and T Cells in MS

### Mechanisms of Infiltration

Under normal physiological conditions, the CNS has been considered an immune privileged environment and consists of a limited number of lymphocytes that cross the blood brain barrier (BBB) (102). However, the revelation of meningeal lymphatic structures emphasized the cross-talk between CNS and peripheral lymphocytes in secondary lymphoid organs (103). The choroid plexus has been identified as the main entry of memory cells into the CNS, which is in the case of T cells mostly mediated by CCR6 (104, 105). The normal human CSF, as is acquired from the arachnoid space by lumbar spinal taps, contains more CD4^+^ Th cells compared to CD8^+^ T cells with central memory characteristics (106–108). The arachnoid space is a continuum with the perivascular space surrounding penetrating arterial and venous structures into the parenchyma (109). Within the brain parenchyma, more CD8^+^ T cells than CD4^+^ Th cells are found, however, their numbers remain low and can be found virtually restricted to the perivascular space (11, 110). These T cells display a phenotype mostly associated with non-circulating tissue resident memory T cells. The perivenular perivascular space has been argued to be the common drainage site of antigens mobilized with the glymphatics flow (111). The exact relationship between memory T cells in the subarachnoid and perivascular space has been poorly identified in terms of replenishment and clonal association.

The BBB is dysfunctional during the early phase of MS, resulting in or is due to local recruitment of pathogenic T and B cells (112). Differential expression of pro-inflammatory cytokines, chemokine receptors and integrins by infiltrating lymphocytes have been argued to mediate disruption of the BBB in MS (104, 113). Myelin-reactive CCR6^+^ and not CCR6^–^ memory Th cells from MS patients not only produce high levels of IL-17, but also IFN-γ and GM-CSF (80). Previous studies mainly focused on the migration of IL-17-producing CCR6^+^ Th cells through the choroid plexus in EAE and *in vitro* human brain endothelial cell layers in MS brain tissues (104, 114). In our recent study, we subdivided these CCR6^+^ memory Th cells into distinct Th17 subsets and found that especially IFN-γ producing Th17.1 (CCR6^+^CXCR3^+^CCR4^–^) cells were capable of infiltrating the CNS, both in *ex vivo* autopsied brain tissues and in *in vitro* transmigration assays (78). The fact that Th17.1 cells have cytotoxic potential and strongly co-express IFN-γ with GM-CSF (78) suggests that these cells are involved in disrupting the permeability of the BBB in MS (115, 116). The impact of CXCR3 on their transmigration capacity is likely the result of binding to the chemokine ligand CXCL10, which is produced by brain endothelial cells and is abundant in the CSF of MS patients (117, 118). Similar observations were made for CXCR3 (T-bet)^+^ B cells (44). CCR6 is also highly expressed on memory B-cell precursors within the Th cell-containing light zone of GCs (119), and on IFN-γ-producing CD8^+^ T cells infiltrating the MS brain (120). This implies that both populations are susceptible to enter the CNS of MS patients. In addition to chemokine receptors and pro-inflammatory cytokines, adhesion molecules such as activated leukocyte cell adhesion molecule (ALCAM) enhance transmigration of pathogenic B and T cell subsets (115, 121, 122). Furthermore, CXCR3 is co-expressed with integrin α4β1 (VLA-4), which allows both B- and T-cell populations to bind to vascular cell adhesion protein 1 (VCAM-1) on brain endothelial cells (123). This is supported by the reducing effects of VLA-4 inhibition on B- and Th17-cell infiltration into the CNS and disease susceptibility in EAE (124). Natalizumab, a monoclonal antibody against VLA-4, is used as an effective second-line treatment for MS (125). Discontinuation of this treatment often results in severe MS rebound effects (126). Hence, the peripheral entrapment of populations like Th17.1 and T-bet^+^ B cells in natalizumab-treated patients (44, 78) probably underlies the massive influx of blood cells causing these effects. The same is true for EBV-reactivated B cells, which are enriched in lesions from MS patients after natalizumab withdrawal (127). A previous gene network approach using several GWAS datasets further highlights the relevance of adhesion molecules on the BBB endothelium for the crossing of T and B cells (128), especially those affected by IFN-γ (115).

### Local Organization and Impact

Both B and T cells accumulate in active white matter lesions of the MS brain (10, 129). In diagnostic biopsy studies, T cell-dominated inflammation is a characteristic of all lesion-types observed (130). Also in post-mortem MS lesions, white matter MS lesions with active demyelination associate with an increase in T cell numbers (10, 129). Although CD4^+^ Th cells are in general outnumbered by CD8^+^ CTLs in brain lesions as investigated in autopsy studies (10), their role as triggers of local pathology should not be overlooked in MS. This is consistent with the enrichment of CD4^+^ Th cells in white matter lesions with active demyelination (10). An abundant number of CD4^+^ Th cells were also visible in pre-active lesion sites, suggesting an involvement of these cells in the early stages of lesion formation (131). Additionally, it was demonstrated that in contrast to CD8^+^ CTLs, brain-associated CD4^+^ Th-cell clonotypes are reduced in MS blood, indicating specific recruitment (as described above) or, alternatively, clonal expansion in the CNS (132). Furthermore, dominant Th-cell clones were undetectable following reconstitution after autologous hematopoietic stem cell transplantation in MS patients, which was not seen for CD8^+^ T cells (133). Interestingly, T-cell clones are shared between CNS compartments within a patient, including CSF and anatomically separated brain lesions (132, 134–137). This suggests that brain-infiltrating T cells bear similar reactivity against local (auto)antigens.

In subsets of MS autopsy cases with acute and relapsing remitting MS, B cells can also be found predominantly in the perivascular space in association with active white matter lesions (10). The role of these perivascular B cells, including T-bet^+^ B cells (44), could be to re-activate (infiltrating) pro-inflammatory CD4^+^ and CD8^+^ T cells to cause MS pathology (Figure 1). Identical B-cell clones have been found in different CNS compartments of MS patients, including the meninges (138, 139). Within the meninges, B- and T cell-rich follicle-like structures have been found that localize next to cortical lesions, presumably mediating progressive loss of neurological function in MS (140, 141). Interestingly, MS brain-infiltrating lymphocytes express and respond to IL-21 (142), the cytokine that drives follicular T- and B-cell responses. Additionally, IFN-γ triggering of B cells promotes ectopic follicle formation in autoimmune mice (16, 45), suggesting that the structures observed in the MS CNS are induced by B cells interacting with IFN-γ-producing T cells. However, the role of IL-17 in this process should not be ruled out, as shown in EAE (143).

Besides mediating migration and organization of pathogenic lymphocytes in the MS brain, cytokines are likely relevant effector molecules. IFN-γ production by Th cells also associates with the presence of demyelinating lesions in the CNS (144–146). IFN-γ, and possibly also GM-CSF, can activate microglia or infiltrated macrophages to cause damage to oligodendrocytes (93, 147, 148). As for B cells, increased production of TNF-α, IL-6, and GM-CSF has been found (48, 87) and we have recently shown that during Tfh-like cultures, IFN-γ drives IgG-producing plasmablasts in MS (44). One could speculate that after their re-activation by IFN-γ-producing Th cells within the meningeal follicles, T-bet^+^ memory B cells rapidly develop into antibody-producing plasmablasts/plasma cells (Figure 1). IFN-γ-induced GC formation promotes the generation of autoantibodies in lupus mice (16, 45). The targeting of B cells and not plasmablasts/plasma cells by clinically effective anti-CD20 therapies in MS, as well as the abundance of oligoclonal bands in MS CSF, at least support the local differentiation of B cells into antibody-secreting cells (48, 149). We argue that IgG secreted by local T-bet-expressing plasmablasts/plasma cells are highly reactive in the MS brain (43, 44, 55), although the (auto)antigen specificity and pathogenicity of such antibodies remain unclear in MS, as well as their contribution as effector molecules to MS pathology.

Several antigenic targets have been proposed to contribute to MS pathology. Next to myelin, which is one of the most intensively studied antigens (150), also EBV antigens are considered as major candidates. EBNA-1 specific IgG antibodies are predictive for early disease activity (151) and are present in CSF from MS patients (152, 153). Some studies imply that reactivated B cells in ectopic meningeal follicles (154, 155) cross-present EBV peptides to activate myelin- and EBNA-1 specific Th cells (6, 156, 157). Whether EBV is detected in the brain or solely recognized in the periphery and how this contributes to local pathology is still a matter of intense debate in the field (127, 158–162). In addition to myelin (150) and EBV (6), other antigenic targets of locally produced IgG and infiltrating T cells have been suggested, such as sperm-associated antigen 16 [SPAG16 (163)], neurofilament light, RAS guanyl-releasing protein 2 [RASGRP2 (4)], αB-crystallin and GDP-l-fucose synthase (135).

## Concluding Remarks

In this review, we have discussed potential triggers and mechanisms through which interacting B and T cells drive the pathogenesis of MS. In our presented model, peripheral B cells escape from tolerance checkpoints as the result of impaired control by chronically exhausted or genetically altered regulatory T cells. Subsequently, B cells interact with IFN-γ-producing effector Th cells in germinal centers of lymphoid organs to create a feedforward loop, after which highly pathogenic subsets break through blood-CNS barriers and, together with infiltrating CD8^+^ CTLs are locally reactivated to cause MS pathology. Although definite proof is still lacking, these pathogenic events are likely mediated by an interplay between persistent infections such as EBV and genetic risk variants. Together, these factors may alter the selection, differentiation and pathogenic features of B- and T-cell subsets. In our view, more in-depth insights into how infections and genetic burden define the CNS-infiltrating potential and antigen specificity of such subsets should be the next step to take in the near future. The development of small molecule therapeutics against subsets driving the disease course would be an effective way of generating clinically relevant benefits without harmful effects in MS patients.

## Author Contributions

JL, LR, and ML designed and wrote the manuscript. ML and JS revised the manuscript.

## Conflict of Interest

JS received speaker/consultancy fee from Biogen, Merck, Novartis, and Sanofi-Genzyme. The remaining authors declare that the research was conducted in the absence of any commercial or financial relationships that could be construed as a potential conflict of interest.
